# Earlier Age at Menopause, Plasma Metabolome, and Risk of Premature Mortality

**DOI:** 10.3390/metabo14110571

**Published:** 2024-10-24

**Authors:** Zeping Yang, Ninghao Huang, Zhenhuang Zhuang, Ming Jin, Ziyi Zhang, Yimin Song, Haoliang Cui, Shan Zhang, Tao Huang, Xiaojing Liu, Nan Li

**Affiliations:** 1 Department of Epidemiology & Biostatistics, School of Public Health, Peking University, Beijing 100191, China; yangzeping1028@pku.edu.cn (Z.Y.); ninghaohuang@bjmu.edu.cn (N.H.); 2211110202@stu.pku.edu.cn (Z.Z.); 2311110188@bjmu.edu.cn (M.J.); 2211210112@stu.pku.edu.cn (Z.Z.); 2116390124@bjmu.edu.cn (Y.S.); huangtao@bjmu.edu.cn (T.H.); 2 Department of Global Health, School of Public Health, Peking University, Beijing 100191, China; pkubruce@bjmu.edu.cn; 3 Department of Health Policy and Management, School of Public Health, Peking University, Beijing 100191, China; shan_zhangmt@pku.edu.cn; 4 Key Laboratory of Epidemiology of Major Diseases, Peking University, Ministry of Education, Beijing 100191, China; 5 Center for Intelligent Public Health, Institute for Artificial Intelligence, Peking University, Beijing 100191, China

**Keywords:** earlier age at menopause, premature mortality, plasma metabolome, mediation analysis

## Abstract

Background/Objectives: Menopause and related metabolites are associated with mortality. However, the relationship between earlier menopause, premature mortality, and the role of metabolomic signatures remains underexplored. This study investigated the association between earlier menopause and premature mortality, and the mediating effect of metabolomic signatures. Methods: This prospective cohort study used data from the UK Biobank, including 33,687 post-menopausal women aged 40–69 years. Age at menopause was obtained from a baseline self-reported questionnaire and analyzed both as a continuous variable and in categories (<40, 40–49, and ≥50 years). Premature mortality was defined as deaths before 75 years. Cox regression was used to estimate hazard ratios (HRs), and elastic net regression identified metabolomic signatures related to menopause age. Mediation analysis was conducted to assess the proportion of the association explained by the metabolomic signature. Results: During a median follow-up of 13.7 years, 1612 cases of premature mortality occurred. Compared to menopause at ≥50 years, earlier menopause (HR 1.17, 95% CI 1.04–1.30) and premature menopause (HR 1.60, 95% CI 1.28–2.00) were associated with higher risks of premature mortality. A metabolomic signature inversely associated with premature mortality (HR per SD increment, 0.79; 95% CI, 0.75–0.83) mediated 13.6% (95% CI, 1.9%–28.3%) of the association between earlier menopause and premature mortality. Conclusions: Earlier menopause is associated with an increased risk of premature mortality, partially mediated by a metabolomic signature related to age at menopause. These findings highlight the importance of metabolomic profiling in understanding menopause and mortality risks.

## 1. Introduction

Menopause is the permanent end of menstruation induced by follicle depletion [1]. The timing of the menopause is often used to indicate a woman’s reproductive health [2,3]. It is a very important biomarker about the loss of fertility and the increased risk for various mid-life diseases [4]. According to previous studies, an earlier age at menopause was a contributor to an increased risk of various diseases, especially cardiovascular diseases [5,6,7]. Based on the data from the World Health Organization European Region, the age-standardized premature mortality rate for the four major noncommunicable diseases was recorded at 379.62 per 100,000 individuals aged between 30 and 69 years in 2015 [8]. Research is needed to reveal the relationship between menopause status and premature mortality.

As far as we could discern, in four studies, an earlier age at menopause has been associated with increased all-cause mortality risks [9,10,11,12]. This result has also been confirmed by meta-analyses based on different cohorts [5,11,13]. Based on the above evidence, an earlier age at menopause could indirectly increase the risk of mortality due to the heightened risks of various diseases. However, there has been no research about the risk of premature mortality associated with an earlier age at menopause. The present study aims to fill the research gap.

Metabolomics is an emerging discipline within life sciences that employs cutting-edge analytic tools combined with advanced statistical approaches to holistically study the metabolome [14]. Emerging studies assess the metabolomic signature related to the disease or health status, to better predict morbidity and mortality [15]. Among various reproductive factors, it has been demonstrated that menopause can modulate the circulating metabolic profiles [16,17,18]. The menopausal status in post-menopausal women was significantly associated with metabolites like C2, Glu, and sarcosine [19], amino acids glutamine, tyrosine, and isoleucine, and serum cholesterol and atherogenic lipoproteins [20]. However, previous studies focused on a limited number of metabolites in serum, and there is a lack of research on a wide range of menopause-related metabolites in plasma and further exploring their overall effect.

Therefore, the primary aim of our study was to investigate the associations between age at menopause and early menopause with premature mortality in the large-scale UK Biobank cohort. Furthermore, using the extensive data of plasma metabolomics profiles, we aimed to identify a menopause age-specific metabolomics signature and explore its potential mediated effect on the risk of premature mortality associated with early menopause.

## 2. Materials and Methods

### 2.1. Study Participants

The UK Biobank is a prospective cohort study with over 502,000 participants across the UK aged 40–69 years when recruited between 2006 and 2010 [21]. At baseline, participants were asked to provide information on sociodemographic factors, family history, lifestyle, and medical history through touchscreen questionnaires [22], and their blood samples were collected between 2007 and 2010 [23]. In this study, we included 33,687 post-menopausal women with complete data on the age at menopause, metabolomics, and mortality. A flowchart of the included participants is shown in Figure S1 in the Supplementary Materials. Ethical approval was obtained from the North West Multi-centre Research Ethics Committee (ref: 21/NW/0157). All participants provided informed consent at recruitment to this study through an electronic signature.

### 2.2. Metabolomic Profiling

A subset of non-fasting baseline plasma samples (aliquot 3) was randomly selected from a large population of individuals (*n* = 118,461) in the UK Biobank. These samples were analyzed using high-throughput nuclear magnetic resonance (NMR) spectroscopy by Nightingale Health Plc (biomarker quantification version 2020). NMR spectroscopy enables precise quantification of metabolites as the signal intensity is directly proportional to the concentrations and the number of particular nuclei [24]. The analysis allowed for simultaneous quantification of 249 metabolomic profiles, including lipoprotein, lipids, amino acids, apolipoproteins, cholesterol, cholesteryl esters, fatty acids, phospholipids, triglycerides, other lipids, free cholesterol, the fluid balance, glycolysis-related metabolites, inflammation, and ketone bodies. Ratios of metabolites (e.g., triglycerides to phosphor glycerides) were excluded since they were not within the scope of our study. A final set of 167 metabolites was available for subsequent analysis, as previously reported [25]. The measure used for plasma metabolomics profiling is described in Method S1 in the Supplementary Materials.

### 2.3. Definition of Earlier Age at Menopause

Participants were asked “How old were you when your periods stopped?” about the age at menopause at baseline. We transformed the continuous age of menopause into three groups (low: <40 years; intermediate: 40–49 years; and high: ≥50 years) [26,27,28]. An earlier age at menopause was defined as menopause occurring before the age of 50 years.

### 2.4. Assessment of Premature Mortality and Covariates

Information regarding mortality and the date of death was obtained by reviewing death certificates, which were held by the National Health Service Information Centre for participants in England and Wales, and the National Health Service Central Register Scotland for participants from Scotland. Person-years at risk were calculated from the date of attending the assessment center until the date of loss to follow-up, the date of death, or 1 January 2023, whichever occurred first. Deaths occurring before the age of 75 were classified as premature mortality [29,30]. The potential covariates included age, race, education, employment, body mass index (BMI), healthy alcohol intake, healthy diet, healthy physical activity status, and menopause hormone therapy. A detailed description of the covariates is presented in Method S2 in the Supplementary Materials.

### 2.5. Statistical Analyses

Continuous variables were presented as their means and standard deviations (SDs) and categorical variables were presented as counts and percentages. We examined the correlations between age at menopause and individual metabolites by calculating Spearman correlation coefficients [25]. A false discovery rate (FDR) of less than 0.05 was considered to indicate statistical significance. The metabolomic signature was computed as the weighted sum of the selected metabolites (original value) with weights (coefficients in the model) using elastic net regression, where age at menopause was the predictor [30,31,32,33]. An elastic net regression model was generated using R version 4.3.1 (R Project for Statistical Computing, https://www.r-project.org/, accessed on 28 July 2023) and the glmnet package version 4.1-8 (https://glmnet.stanford.edu/, accessed on 28 July 2023). We classified metabolomic signatures into low (bottom quartile), intermediate (quartiles 2–3), and high (top quartile) categories.

Then, we conducted Kaplan–Meier survival analysis to plot the cumulative incidence of premature mortality in age at menopause and metabolomic signature groups. The log-rank test was used to assess the statistical significance of observed disparities in survival distributions. Furthermore, we used Cox proportional hazards models to estimate hazard ratios (HRs) and 95% CIs of associations between age at menopause and metabolomic signature with premature mortality. The Cox models were validated using the proportional hazards assumption test (Method S3 and Figure S4 in Supplementary Materials). Each SD (5.13 years) increment of menopausal age and metabolomic signature for premature mortality is displayed. We also estimated the associations between age at menopause and cause-specific premature mortality from cancer, chronic liver disease, type 2 diabetes, hypertension, cardiovascular disease, chronic kidney disease, and all other causes. Information on the codes for diseases was shown in Table S1 in the Supplementary Materials. The population attributable risk percentage (PAR%) was calculated to estimate the proportion of premature mortality that theoretically would not have occurred if all participants were in the high age at menopause group or high metabolomic signature group. Cox models were employed without any adjustments (Model 1), adjusting for age, race, education, employment, BMI, healthy alcohol intake, healthy diet, healthy physical activity, and menopause hormone therapy (Model 2), and further mutually adjusting for age at menopause and metabolomic signature (Model 3).

We employed restricted cubic spline (RCS) regression (Method S4 in Supplementary Materials) to model the relationships between age at menopause, metabolomic signature, and the risk of premature mortality, as well as their potential non-linear associations. In an additional sensitivity analyses, we evaluated associations of menopause and related metabolomic signatures with premature mortality before 65 and 70 years of age. The mediating role of the metabolomic signature was tested through mediation analyses with the R “mma” package (Method S5 in Supplementary Materials) [31,32]. The proportions of missing data for covariates (age, race, education, employment, BMI, healthy alcohol intake, healthy diet, healthy physical activity, and menopause hormone therapy) were all less than 5%, and no imputation methods were necessary [33]. All *p*-values were reported as two-sided tests, with significance defined as *p* < 0.05. All analyses were conducted using the R software (Version 4.3.1, R Core Team) [34].

## 3. Results

### 3.1. Population Characteristics

Table 1 displays the characteristics of the study population and its comorbidity statuses by age at menopause categories. Of the eligible participants, 21,157 (62.80%) individuals experienced menopause ≥50 years, 11,148 (33.09%) individuals experienced menopause at 40–49 years, and 1382 (4.10%) individuals experienced menopause at <40 years. Participants who had premature menopause tended to be younger, have a higher BMI, a lower education level, a less healthy alcohol intake, and a less healthy diet, and exhibited lower levels of healthy physical activity, a higher proportion of menopause hormone therapy, and a higher incidence of comorbidities related to mortality (e.g., cardiovascular disease, chronic kidney disease, and liver disease). In terms of employment status, participants in the age groups of ≥50 years and <40 years were more likely to be retired.

### 3.2. Identification of the Menopause-Related Metabolomic Signature

**Figure 1:**
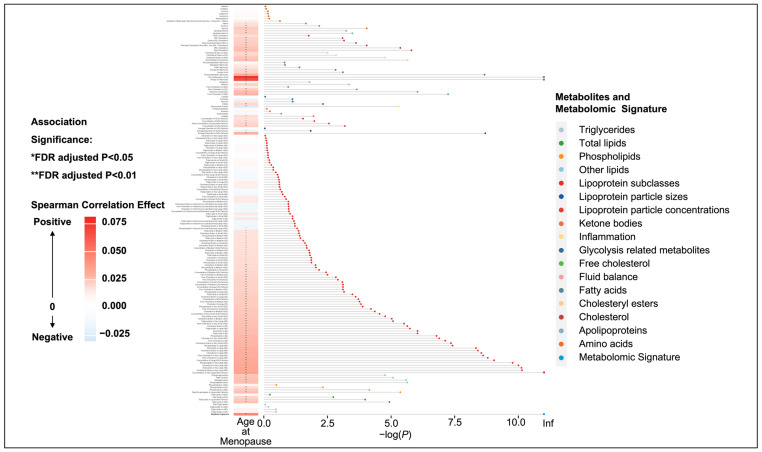
Heatmap and dot–bar plot for the association between age at menopause and all metabolomic biomarkers. The *x*-axis represents the false-discovery rate (FDR)-adjusted *p*-value after negative logarithmic transformation, and the *y*-axis represents the metabolites, sorted by their groups and the significance of associations. *: FDR-adjusted *p*-value < 0.05; **: FDR-adjusted *p*-value < 0.01.

A significant correlation with age at menopause was observed for 106 metabolites, accounting for 63.47% of the total (Figure S2 in Supplementary Materials). Significant associations were found between age at menopause and lipoproteins, lipids, fatty acids, and amino acids (Figure 1). The metabolomic signature for age at menopause included 163 metabolites that were significantly associated with age at menopause in the elastic net regression (Table S2 in Supplementary Materials). The top 25 metabolites identified according to the regression coefficients mainly belonged to lipoprotein, followed by free cholesterol, fatty acids, and other lipids. The composition and weight of individual metabolites included in this metabolomic signature closely resemble the 167 metabolites, with the exclusion of one lipoprotein (triglycerides in VLDL), one triglyceride (free cholesterol in medium HDL), and two types of cholesterol (VLDL cholesterol and clinical LDL cholesterol). Compared to the others, several metabolites exhibited significantly stronger associations with age at menopause, including lipids, lipoprotein, and fatty acids. The metabolomic signature was significantly correlated with age at menopause (r = 0.05; *p* = 2.2 × 10^−16^).

### 3.3. Associations of Metabolomic Signature at Earlier Age of Menopause with Premature Mortality Risk

**Figure 2:**
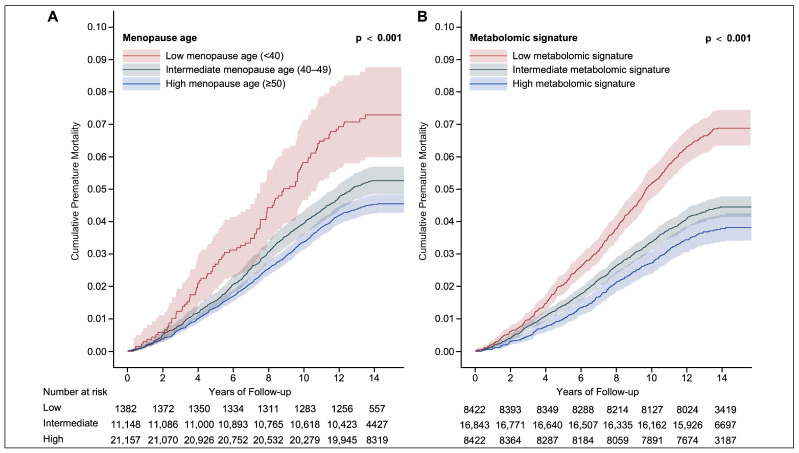
Cumulative premature mortality by age at menopause and metabolomic signature groups. Cumulative premature mortality stratified by age at menopause (**A**) and metabolomic signature (**B**) of participants in the UK Biobank. Number at risk represents the number of participants at risk at a specific time point.

The incidence levels per 100,000 person-years were 332.33, 385.74, and 543.96 in the ≥50 years, 40–49 years, and <40 years groups, respectively. The numbers at risk for each 2-year follow-up period are presented in Figure 2. Based on the Kaplan–Meier curves in Figure 2A, participants with earlier menopause had a higher risk of incidence of premature mortality when compared with participants whose age at menopause was ≥50 years. A similar trend was observed in the categories of the metabolomic signature (Figure 2B). The associations of different categories of the metabolomic signature with premature mortality risk are presented in Table S3 in the Supplementary Materials. Compared to the participants in the high metabolomic signature group, those in the low and intermediate groups had 65% (adjusted HR [aHR] 1.65, 95% CI 1.42–1.91) and 18% (aHR 1.18, 95% CI 1.03–1.35) higher risks of premature mortality after fully adjusting for confounders (Model 3), respectively. There was an inverse relationship of the metabolomic signature level and premature mortality risk, with the *p*-value for the trend <0.001.

As shown in Table 2, age at menopause had a significant inverse association with the risk of premature mortality in Model 2 (aHR 0.98, 95% CI 0.97–0.99). The higher risk of premature mortality among earlier menopause remained clear (40–49 years: aHR 1.17, 95% CI 1.04–1.30, *p* < 0.001; <40 years: aHR 1.60, 95% CI 1.28–2.00, *p* < 0.001) in Model 2. The 5.13-year increase in age at menopause was related to an 11% (7–16%) lower risk of premature mortality. The PAR% for premature mortality associated with the age at menopause was 7.20% (3.32–11.10%). In Model 2, the 5.13-year increase in age at menopause was related to 3% (0–5%), 7% (3–10%), 9% (3–14%), and 2% (1–3%) lower risks of premature mortality caused by cardiovascular disease, chronic kidney disease, chronic liver disease, and all other causes of death, respectively (Table S4 in Supplementary Materials). In Model 2, an inverse association with premature mortality was also observed in the continuous metabolomic signature (aHR 0.70, 95% CI 0.65–0.76). The higher risk of premature mortality among lower metabolomic signatures remained clear (intermediate: aHR 1.18, 95% CI 1.03–1.35, *p* < 0.001; low: aHR 1.65, 95% CI 1.42–1.91, *p* < 0.001), with the *p*-value for the trend <0.001. The SD increase in metabolomic signature was related to a 21% (17–25%) lower risk of premature mortality. The PAR% for premature mortality associated with the metabolomic signature was 19.90% (11.30–28.40%). In a sensitivity analysis using varying cutoff points for premature death (65 and 70 years of age), associations of age at menopause and the metabolomic signature with premature mortality remained consistent (Table S5 in Supplementary Materials).

RCS visualized the relationships of age at menopause and metabolomic signature with the incidence of premature mortality (Figure S3 in Supplementary Materials). As the age at menopause and metabolomic signature decreased, the risk of premature mortality remained relatively flat until around age 50 and a metabolomic signature of 11.39, respectively, after which it began to increase rapidly (age at menopause: *p* for non-linearity = 0.164, *p* for linearity <0.001; metabolomic signature: *p* for non-linearity <0.001, *p* for linearity <0.001).

### 3.4. Mediation Analyses of the Menopause–Premature Mortality Association

**Figure 3:**
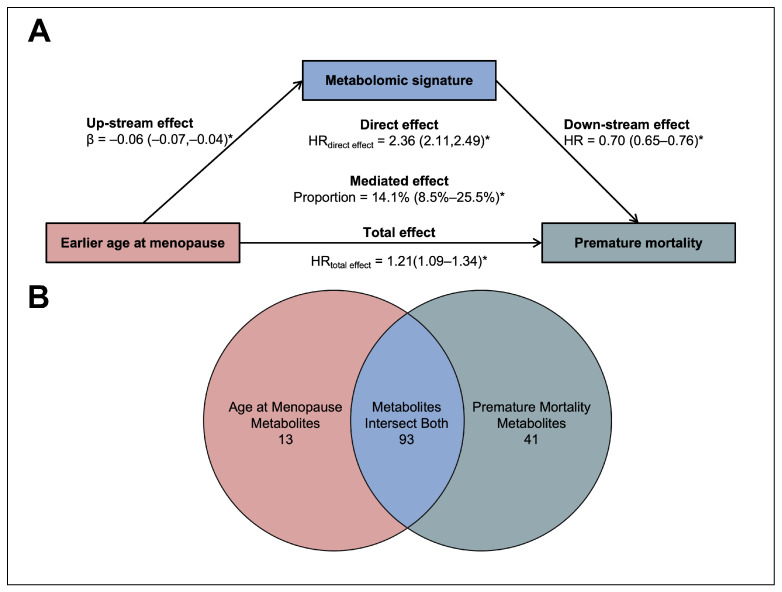
Role of plasma metabolomics in the association of age at menopause with premature mortality. (**A**) Mediation effect of metabolomic signature on the earlier age at menopause–premature mortality association. After adjusting for age, race, education, employment, BMI, healthy alcohol intake, healthy diet, healthy physical activity, and menopause hormone therapy, the total and direct effects of an earlier age at menopause on the risk of premature mortality and the mediated proportion of the metabolomic signature are shown, with 95% CIs for the effect estimates provided in parentheses. * *p*-value < 0.05. (**B**) Venn diagram for metabolites correlating with age at menopause and premature mortality. The diagram illustrates the number of metabolites associated with an earlier age at menopause (13 metabolites) or with an premature mortality (41 metabolites), and those intersecting both conditions (93 metabolites).

As shown in Figure 3A, the metabolomic signature showed partial mediating effects on the association between earlier menopause and premature mortality, with the significant proportion (95% CI) of the mediation effect being 13.6% (1.9–28.3%) after adjusting for covariates. In addition, we observed that the association between an earlier age at menopause and premature mortality risk reduced after further adjusting for the metabolomic signature (aHR 1.19, 95% CI 1.07–1.32). Figure 3B presents the overlap of metabolites associated with an earlier age at menopause and premature mortality. Details of these metabolites are given in Table S6 in the Supplementary Materials.

## 4. Discussion

Leveraging data from a comprehensive, population-based, prospective cohort, our study identified a metabolomic signature linked to menopausal age and its correlation with premature mortality risk. Our research revealed the potential inverse relationship between an earlier age at menopause and the risk of premature mortality, mediated by our newly developed metabolomic signature. These findings lead us to introduce a new metabolomic signature related to menopausal age and to elucidate the link between early menopause and a heightened premature mortality risk.

Previous studies revealed that women’s menopausal status was significantly associated with metabolites like C2, Glu, and sarcosine [19], amino acids glutamine, tyrosine, and isoleucine, and serum cholesterol and atherogenic lipoproteins [20]. Our findings were consistent with previous studies as total free cholesterol contributed the greatest loadings to the menopause-related metabolomic signatures. While previous studies focused on a limited number of metabolites, our study encompassed a wide range of metabolites in plasma. It is worth noting that most of the metabolites related to menopause in previous studies were analyzed in serum. However, our study explored plasma metabolites and, for the first time, identified a unique metabolomic signature. Furthermore, in numerous studies, an earlier age at menopause was associated with increased all-cause mortality risks [9,10,11,12]. According to the Global Burden of Disease Study 2019, ischemic heart disease and stroke are crucial components of premature mortality, especially in older individuals [35]. Premature ovarian insufficiency [36], previously termed premature menopause or primary ovarian failure [37], is the early cessation of menses before age 40 due to ovarian dysfunction. A study indicated that premature ovarian insufficiency might be associated with increased risks of ischemic heart disease and stroke [38,39]. Our results are consistent with prior research linking an earlier age at menopause to premature mortality due to heightened ischemic heart disease and stroke risks.

Another notable finding was that participants with a low metabolomic signature faced a 65% increased risk of premature mortality. This aligned with the detrimental impact of earlier menopause on mortality [9,10,11,12]. We also found that approximately 14% of the harmful effect of earlier menopause on the risk of premature mortality was mediated by the metabolomic signature. Our findings showed that the top 25 metabolites in the menopausal age-related metabolomic signature mainly belonged to lipoprotein, free cholesterol, and fatty acids. In previous research, it was shown that a menopausal status accompanied by hormonal changes may contribute to dysregulation of various components of lipid metabolism, e.g., lipoproteins, fatty acid, and cholesterol [16,17,40,41], which is consistent with our findings. According to previous studies, dysregulation of cholesterol is a pivotal factor leading to atherosclerosis [42,43], a condition characterized by the narrowing of arteries due to the progressive accumulation of cholesterol as arterial plaques [43]. This arterial constriction can result in serious health outcomes such as stroke, hypertension, heart failure, and heart attacks [42]. Atherosclerosis has been a primary contributor to cardiovascular disease and the leading causes of mortality globally [35,42]. Therefore, metabolic dysregulation may contribute to a higher risk of premature mortality potentially via cholesterol dysregulation. Those potential pathways were also reflected by metabolites identified in our study; for instance, total free cholesterol contributed the greatest loadings to the menopause-related metabolomic signatures. Our study provides novel insights into the mechanistic links between the age at menopause and premature mortality.

Previous studies usually applied the self-reported age at menopause, which can be less precise, especially in older women who are further from their menopausal phase [7,28]. In this research, we uniquely derived a metabolomic signature by applying elastic net regression to the age of menopause based on 167 metabolites, effectively mitigating potential inaccuracies inherent in self-reported menopausal data. Indeed, our findings highlighted a more pronounced association between the metabolomic signature and the risk of premature mortality compared to the self-reported age at menopause.

This investigation presents notable advantages. Primarily, we conducted a large-scale prospective cohort study utilizing the data of the UK Biobank, spanning a relatively long follow-up duration. Additionally, the presence of diverse covariates, encompassing demographics, physical attributes, lifestyle habits, and metabolic factors, facilitated detailed adjustments for conceivable confounders. Furthermore, our study encompassed a wide range of metabolites in plasma and identified a distinct metabolomic signature for the first time, while previous studies focused on a limited number of menopause-related metabolites in serum. Last but not least, a linear trend between the age at menopause and premature mortality was observed from an RCS analysis, also indicating a dose–response relationship between earlier menopause and premature mortality.

It is also imperative to understand the findings within the scope of certain constraints. Initially, the research undertook a retrospective evaluation of the data of the UK Biobank, meaning the confounders were contingent on the dataset’s inherent variables. This poses the risk of undetected or unquantified biases influencing the correlation between premature menopause and premature mortality. Furthermore, detailed information on medical usage after menopause, which might mitigate the risk of menopause-related mortality, was unavailable. We additionally included menopause hormone therapy as a covariate; however, the results were nearly unchanged. Furthermore, the information on menopause age and certain covariates was from self-reported questionnaires at baseline and ascertained by one-time measurement, which were likely to induce potential inaccuracies or misclassifications due to recall bias. In particular, we could not verify whether the duration of menopause exceeded the 12-month benchmark as delineated by the WHO’s menopause criteria [44]. Such self-reported inconsistencies can induce regression attenuation bias, thereby potentially diminishing the actual effect size. The third point of consideration is that the low response rate in the cohort and healthy volunteer bias may have contributed to an underestimation of the impact of menopause on premature mortality [45], which needs to be further assessed in future studies. Fourth, although NMR spectroscopy can precisely detect a wide range of metabolites at the same time, a challenge remains in improving the signal sensitivity. Lastly, given that the study predominantly comprised White British participants, the findings’ applicability might be constrained when considering other ethnicities.

## 5. Conclusions

Drawing upon individual data from the comprehensive UK Biobank study, we have identified a metabolomic signature that characterizes the age of menopause and have found an association between the metabolomic signature and premature mortality. These observations have significant public health implications, offering both a more refined appraisal of menopausal status and fresh understandings of the interplay between earlier menopausal onset and heightened premature mortality risk. Further investigations are imperative to elucidate the exact mechanisms at play and develop interventions to mitigate the onset of early menopause, potentially reducing associated premature mortality risks.

## Figures and Tables

**Table 1:** The characteristics of participants in the UK Biobank Study according to age at menopause (*n* = 33,687).

Characteristic	Age at Menopause			*p*-Value ^a^
	≥50 Years (*n* = 21,157)	40–49 Years (*n* = 11,148)	<40 Years (*n* = 1382)	
Age at menopause, mean (SD), y	52.68 (2.44)	45.74 (2.76)	35.21 (3.90)	<0.001
Age, mean (SD), y	60.74 (4.71)	59.07 (6.31)	58.46 (7.24)	<0.001
Body mass index, mean (SD), kg/m^2^	27.05 (4.93)	27.17 (5.10)	27.68 (5.24)	<0.001
Race, White British, No. (%)	19,122 (90.38)	9838 (88.24)	1214 (87.84)	<0.001
Education, no. (%)				<0.001
Work-related practical qualifications	900 (4.25)	611 (5.48)	104 (7.53)	
Lower secondary education	5582 (26.38)	3027 (27.15)	398 (28.80)	
Upper secondary education	2267 (10.72)	1114 (9.99)	114 (8.25)	
Higher education	8034 (37.97)	3813 (34.20)	361 (26.12)	
None of the above	4175 (19.73)	2462 (22.08)	387 (28.00)	
Employment, no. (%)				<0.001
In paid employment or self-employed	9135 (43.18)	5195 (46.60)	577 (41.75)	
Not in paid employment	1418 (6.70)	958 (8.59)	150 (10.85)	
Retired	10,425 (49.27)	4886 (43.83)	642 (46.45)	
Healthy alcohol intake, no. (%)	9872 (46.66)	4917 (44.11)	539 (39.00)	<0.001
Healthy diet, no. (%)	3884 (18.36)	1900 (17.04)	231 (16.71)	0.011
Healthy physical activity status, no. (%)	14,465 (68.37)	7371 (66.12)	871 (63.02)	<0.001
Menopause hormone therapy, no. (%)	8896 (42.05)	5437 (48.77)	1001 (72.43)	<0.001
Comorbidities related to mortality				
Type 2 diabetes, no. (%)	777 (3.67)	460 (4.13)	82 (5.93)	<0.001
Hypertension, no. (%)	5874 (27.76)	3028 (27.16)	428 (30.97)	0.011
Cardiovascular disease, no. (%)	1120 (5.29)	712 (6.39)	150 (10.85)	<0.001
Chronic kidney disease, no. (%)	21 (0.10)	24 (0.22)	6 (0.43)	0.001
Chronic liver disease, no. (%)	23 (0.11)	16 (0.14)	3 (0.22)	0.304
Cancer, No. (%)	2951 (13.95)	1743 (15.64)	264 (19.10)	<0.001

^a^ *p*-values calculated using Wilcoxon rank sum test, Pearson’s chi-squared test, and Fisher’s exact test. Abbreviation: SD, standard deviation.

**Table 2:** Association of age at menopause and metabolomic signature with premature mortality risk.

	Case/Person-Years	Model 1 ^a^ Coeff, 95% CI	Model 2 ^a^ Coeff, 95% CI	Model 3 ^a^ Coeff, 95% CI
Age at menopause (continuous), HR	1612/449,595	0.98 (0.97, 0.98)	0.98 (0.97, 0.99)	0.98 (0.97, 0.99)
Age at menopause (categorical) ^b^, HR				
High	940/282,851	Reference	Reference	Reference
Intermediate	573/148,544	1.16 (1.05, 1.29)	1.17 (1.04, 1.30)	1.15 (1.03, 1.28)
Low	99/18,200	1.64 (1.34, 2.02)	1.60 (1.28, 2.00)	1.57 (1.25, 1.96)
Each SD increment ^c^	NA	0.88 (0.84, 0.92)	0.89 (0.84, 0.93)	0.89 (0.85, 0.94)
PAR ^d^, %	NA	7.84 (3.56, 12.10)	7.20 (3.32, 11.10)	6.42 (2.63, 10.20)
*p* for trend	NA	<0.001	<0.001	<0.001
Metabolomic signature (continuous), HR	1612/449,595	0.65 (0.60, 0.70)	0.70 (0.65, 0.76)	0.71 (0.65, 0.76)
Metabolomic signature (categorical) ^e^, HR				
High	313/113,393	Reference	Reference	Reference
Intermediate	734/225,400	1.18 (1.03, 1.35)	1.18 (1.03, 1.35)	1.18 (1.02, 1.35)
Low	565/110,800	1.84 (1.61, 2.12)	1.65 (1.42, 1.91)	1.63 (1.41, 1.89)
Each SD increment	NA	0.75 (0.71, 0.79)	0.79 (0.75, 0.83)	0.79 (0.75, 0.84)
PAR ^d^, %	NA	23.00 (15.20, 30.90)	19.90 (11.30, 28.40)	19.40 (11.30, 27.50)
*p* for trend	NA	<0.001	<0.001	<0.001

^a^ Model 1 was not adjusted; Model 2 was adjusted for age, race, education, employment, body mass index, healthy alcohol intake, healthy diet, healthy physical activity, and menopause hormone therapy; in Model 3, we further conducted mutual adjustment. We included both age at menopause and the menopause-related metabolomic signature simultaneously in this model to examine the association independence. ^b^ Age at menopause was categorized into low (<40 years), intermediate (40–49 years), and high (≥50 years) groups. ^c^ Each SD increment was equal to a 5.13-year increase in menopausal age. ^d^ The percentage of premature mortality theoretically attributable to age at menopause and metabolomic signature among participants included in this study. ^e^ Metabolomic signature was categorized into low (bottom quartile), intermediate (quartiles 2–3), and high (top quartile) groups. Abbreviations: Coeff, coefficients; 95% CI, 95% confidence interval; HR, hazard ratio; PAR%, population attributable risk percentage.

## Data Availability

N. Li had full access to all of the data in this study and takes responsibility for the integrity of the data and the accuracy of the data analysis. For access, please email: linan01@pku.edu.cn. The data are not publicly available due to [strict confidentiality and privacy protections for the participants].

## Supplementary Materials

The following supporting information can be downloaded at https://www.mdpi.com/article/10.3390/metabo14110571/s1, Method S1: Metabolomics Profiling Measurement; Method S2: Detailed Description of Covariates; Method S3: Proportional Hazards Assumption Test for Cox Models; Method S4: Restricted Cubic Spline Analysis; Method S5: Mediation Analysis Test for Cox Models; Figure S1: Flowchart of Study Participants; Figure S2: Histogram for the frequency of metabolites in different categories; Figure S3: The Relationship between Risk of Premature Mortality with Age at Menopause and Metabolomic Signature in the Restricted Cubic Spline Analyses; Figure S4: Schoenfeld Residuals Plot for the Proportional Hazards Assumption of Cox Regression Model; Table S1: Codes for Diseases Related to Cause-specific Premature Mortality in the UK Biobank; Table S2: Coefficient of Variation of the Metabolites Analyzed in the Study; Table S3: Risk of Premature Mortality according to Different Categories of Metabolomic Signature Status; Table S4: Cox Regression Analyses of Age at Menopause and Cause-specific Premature Mortality; Table S5: Associations of Age at Menopause and Metabolomic Signature with Premature Mortality by Varying Age Cut-off Points; Table S6: Metabolites Exclusively Correlated with Age at Menopause, Premature Mortality, and Their Intersection. Refs. [46,47,48,49,50,51,52,53,54,55,56,57,58,59] are cited in Supplementary Materials.
