# Supplementary material for: Earlier Age at Menopause, Plasma Metabolome, and Risk of Premature Mortality
Source: Metabolites. 2024 Oct 24;14(11):571. doi: 10.3390/metabo14110571 (PMC11596455; doi:10.3390/metabo14110571)
Supplement: Supplementary file 1 [file metabolites-14-00571-s001.zip › metabolites-3238888-supplementary.pdf]

Supplementary Materials

Method S1. Metabolomics Profiling Measurement
Method S2. Detailed Description of Covariates
Method S3. Proportional Hazards Assumption Test for Cox Models
Method S4. Restricted Cubic Spline Analysis
Method S5. Mediation Analysis Test for Cox Models
Figure S1. Flowchart of Study Participants
Figure S2. Histogram for the frequency of metabolites in different categories
Figure S3. The Relationship between Risk of Premature Mortality with Age at Menopause and Metabolomic Signature in the Restricted Cubic Spline Analyses
Figure S4. Schoenfeld Residuals Plot for the Proportional Hazards Assumption of Cox Regression Model
Table S1. Codes for Diseases Related to Cause-specific Premature Mortality in the UK Biobank
Table S2. Coefficient of Variation of the Metabolites Analyzed in the Study
Table S3. Risk of Premature Mortality according to Different Categories of Metabolomic Signature Status
Table S4. Cox Regression Analyses of Age at Menopause and Cause-specific Premature Mortality
Table S5. Associations of Age at Menopause and Metabolomic Signature with Premature Mortality by Varying Age Cut-off Points
Table S6. Metabolites Exclusively Correlated with Age at Menopause, Premature Mortality, and Their Intersection
References

Method S1. Metabolomics Profiling Measurement

Samples were collected at baseline from 22 assessment centers across the UK between 2007 and 2010. The blood sample handling and storage protocol has been described previously.[1] Metabolomic profiling in plasma was conducted in Finland from 2019 to 2020 using six NMR spectrometers. The metabolomic profiling protocol was described elsewhere.[2] To be specific, cryopreserved plasma samples were thawed and centrifuged, and the supernatant was mixed with phosphate buffer. The samples were then loaded onto a cooled sample changer, and 2 NMR spectra of each plasma sample were recorded using a 500 MHz NMR spectrometer (Bruker). Accredited quality control was done during the whole process to eliminate systemic and technical variance, and only samples and biomarkers that underwent the quality control process were stored in the UK Biobank dataset. Finally, the metabolic metrics were quantified using the Nightingale Health biomarker quantification library 2020, including 168 metrics presented at absolute levels (ie, fatty acids, glycolytic metabolites, ketone bodies, amino acids, lipids, and lipoproteins) and 81 metrics presented as ratio values.

Method S2. Detailed Description of Covariates

Age and body mass index were included as a continuous variable. Race was divided into White British or others, which included Asian/Asian British, Black/Black British, Chinese, Mixed, and others. Education was divided into work-related practical qualifications, lower secondary education, upper secondary education, higher education, or none of the above. Employment status was divided into in paid employment or self-employed, not in paid employment, or retired. Healthy alcohol intake was defined as 0<alcohol intake<14g/day.[3] Healthy diet score (ranging from 0 to 5) was computed based on the following criteria: consuming a minimum of 4 tablespoons of vegetables per day; consuming at least 3 pieces of fruit per day; consuming fish at least twice a week; limiting unprocessed red meat intake to no more than twice weekly and restricting processed meat consumption to no more than twice weekly. Each criterion met was assigned a score of 1. Healthy diet status was defined as score ≥4.[4] Physical activity was assessed using the short-form International Physical Activity Questionnaire (IPAQ), which captured information on the duration and frequency of walking, moderate-intensity, and vigorous-intensity physical activities. Healthy physical activity status was defined as ≥150 min/week moderate or ≥75 min/week vigorous or 150 min/week mixed (moderate + vigorous) activity.[5] Information of menopause hormone therapy was collected through the question in baseline questionnaire: “Have you ever used hormone replacement therapy?” (UK Biobank Data-Field 2814).

Method S3. Proportional Hazards Assumption Test for Cox Models

We conducted a thorough assessment of the proportional hazards (PH) assumption for age at menopause included in our Cox proportional hazards models. The testing was performed using Schoenfeld residuals, which were plotted against time to visually inspect for any trends. We further employed the Grambsch-Therneau test to quantitatively assess the constancy of hazard ratios over time.[6] The detailed residual plots and statistical test results were provided in eFigure 4.

Method S4. Restricted Cubic Spline Analysis

Restricted cubic spline (RCS) analyses are smooth, piecewise polynomial functions used to model the association between a continuous variable and an outcome without assuming a specific form about their association.[7,8] Multivariable RCS models in our study were employed using the Cox proportional hazards regression with 3 knots set at the 10th, 50th, and 90th percentiles, where the 3 knots were determined based on the lowest Akaike information criterion.[7,9] Those models estimated the nonlinear associations between age at menopause and metabolomic signature with premature mortality. Adjustments were made for confounders including age, race, education, employment, BMI, healthy alcohol intake, healthy diet, healthy physical activity, and menopause hormone therapy. RCS modeling was conducted using R version 4.3.1 (R Project for Statistical Computing, https://www.r-project.org/) and the rms package version 6.7-1 (https://hbiostat.org/r/rms/).[10] RCS curves were visualized with the ggplot2 package version 3.4.4 (https://ggplot2.tidyverse.org/). Additionally, the P value for nonlinearity was calculated using the anova function from the stats package version 4.4.0.

Method S5. Mediation Analysis Test for Cox Models

Age at menopause was recorded during initial assessment visit from 2006 to 2010, and it was recalled from retrospective way. The samples were collected at baseline from 22 assessment centers across the UK between 2007 and 2010. The person-time of mortality was prospectively calculated from baseline. We can therefore infer that the plasma metabolome levels mediate the association between age at menopause and premature mortality from the perspective of time sequence. In the assumptions about the model of mediation analysis, there are generally two settings for mediation analysis. One is based on linear models [11,12], and the other is based on the counterfactual framework [13-15]. We used the R "mma" package (n means the time of resampling in calculating the indirect effects and it equals 20; n2 means the number of times of bootstrap resampling and it equals 100) for the mediation analysis.

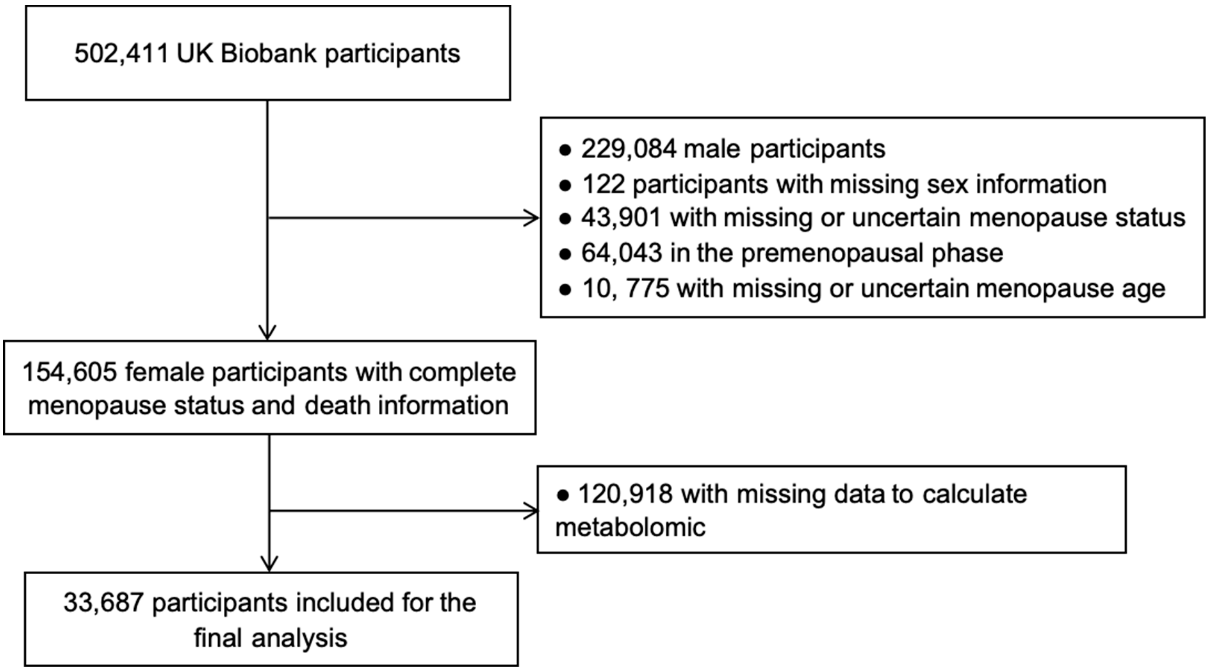

Figure S1. Flowchart of Study Participants

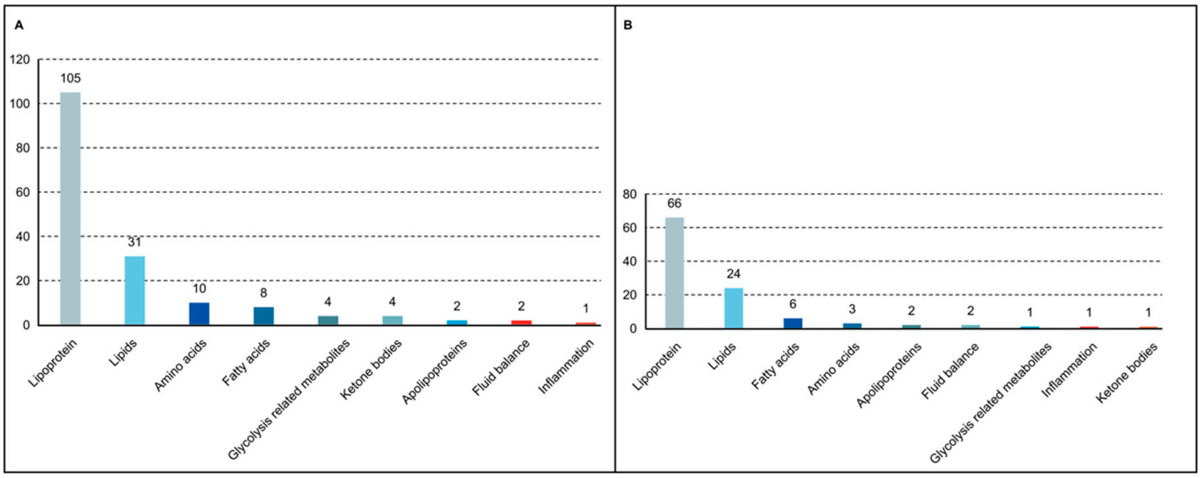

Figure S2. Histogram for the Frequency of Metabolites in Different Categories

(A) The initial 167 metabolites in the UK Biobank that were used to analyze their correlation with age at menopause by Spearman correlation analyses. (B) The 106 metabolites that significantly correlated with age at menopause.

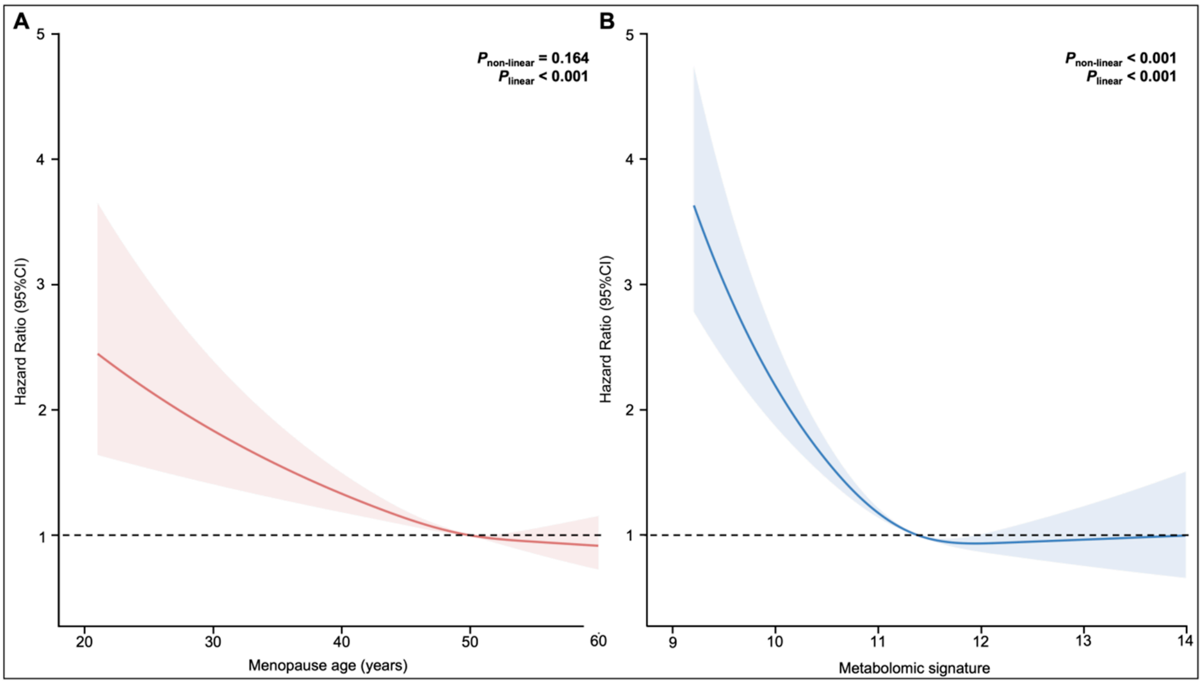

Figure S3. The Relationship between the Risk of Premature Mortality with Age at Menopause and Metabolomic Signature in the Restricted Cubic Spline Analyses

The line represented the hazard function, and the shaded area represented the 95% CIs. Splines adjusted for age, race, education, employment, BMI, healthy alcohol intake, healthy diet, healthy physical activity, and menopause hormone therapy. Non-linear relationships were estimated using RCS and linear relationships were estimated using Cox-proportional hazards regression analysis. (A) RCS plot with multiple adjusted HRs (95% CI) for premature mortality associated with age at menopause. (B) RCS plot with multiple adjusted HRs (95% CI) for premature mortality associated with metabolomic signature.

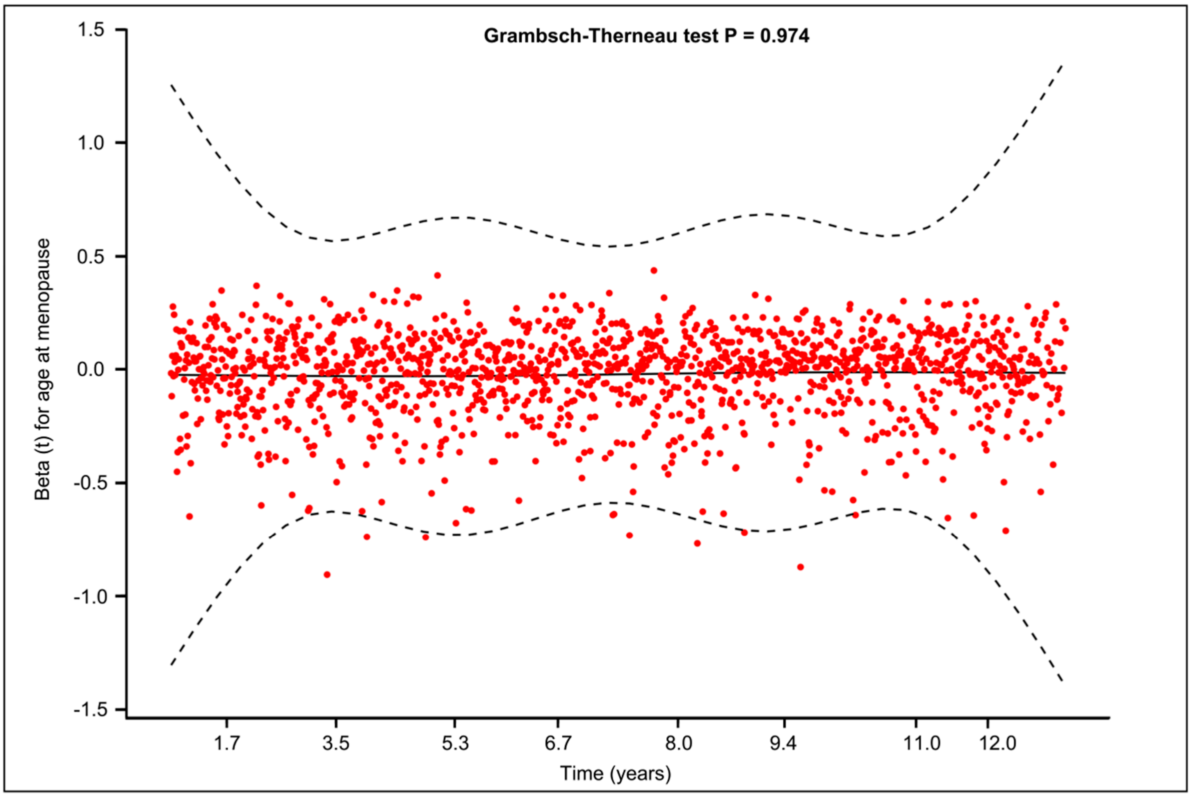

Figure S4. Schoenfeld Residuals Plot for the Proportional Hazards Assumption of Cox Regression Model

Each red dot represents a residual, the solid black line is the fitted residual line, and the dashed black lines represent the 95% confidence interval. The Grambsch-Therneau test yielded a P-value of 0.974, indicating that the proportional hazards assumption is not violated, and the effect of age at menopause on premature mortality remains constant over time.

Table S1. Codes for Diseases Related to Cause-specific Premature Mortality in the UK Biobank

Diseases	ICD-10	ICD-9	Self-report
**Cancer**	C00-C97		
**Chronic liver disease**			
Alcoholic fibrosis and sclerosis	K70.2		
Alcoholic cirrhosis	K70.3		
Alcoholic hepatic failure	K70.4		
Hepatic fibrosis and sclerosis	K74.0, K74.1, K74.2		
Other and unspecified cirrhosis	K74.6		
Portal hypertension	K76.6		
Oesophageal varices	I85		
**Type 2 diabetes**	E11-E14	250	1220, 1223
**Hypertension**	I10-I15	401, 405	1065, 1072
**Cardiovascular disease**			
Coronary heart disease	I20-I25	410-414	1074, 1075
Heart Failure	I50	428	1076
Stroke	I160-I164	430-438	1081, 1086, 1491, 1583
Peripheral artery disease	I70, I73	443	1067
Atrial fibrillation	I48	427	1471
**Chronic kidney disease**			
High risk chronic kidney disease	N18	585	1192, 1193, 1194
Low risk chronic kidney disease	I12, I13, N17-N19, Z49, Z94		

Table S2. Coefficients of Variation of the Metabolites Analyzed in the Study

Rank[a]	Field ID	Metabolites	Units	Group	Coefficients
1	23419	Total Free Cholesterol	mmol	Free cholesterol	1.80E+00
2	23511	Phospholipids in Small VLDL	mmol	Lipoprotein subclasses	-1.47E+00
3	23502	Concentration of Medium VLDL Particles	mmol	Lipoprotein subclasses	-1.18E+00
4	23498	Cholesterol in Large VLDL	mmol	Lipoprotein subclasses	1.17E+00
5	23550	Triglycerides in Small LDL	mmol	Lipoprotein subclasses	1.17E+00
6	23562	Cholesteryl Esters in Large HDL	mmol	Lipoprotein subclasses	1.14E+00
7	23449	Linoleic Acid	mmol	Fatty acids	1.14E+00
8	23518	Phospholipids in Very Small VLDL	mmol	Lipoprotein subclasses	-1.10E+00
9	23445	Omega-6 Fatty Acids	mmol	Fatty acids	-1.08E+00
10	23535	Free Cholesterol in Large LDL	mmol	Lipoprotein subclasses	-1.03E+00
11	23553	Phospholipids in Very Large HDL	mmol	Lipoprotein subclasses	-1.03E+00
12	23528	Free Cholesterol in IDL	mmol	Lipoprotein subclasses	1.00E+00
13	23575	Cholesterol in Small HDL	mmol	Lipoprotein subclasses	-9.99E-01
14	23577	Free Cholesterol in Small HDL	mmol	Lipoprotein subclasses	-9.84E-01
15	23573	Total Lipids in Small HDL	mmol	Lipoprotein subclasses	8.90E-01
16	23437	Phosphatidylcholines	mmol	Other lipids	-8.27E-01
17	23427	Total Concentration of Lipoprotein Particles	mmol	Lipoprotein particle concentrations	8.11E-01
18	23422	Free Cholesterol in HDL	mmol	Free cholesterol	-7.93E-01
19	23574	Phospholipids in Small HDL	mmol	Lipoprotein subclasses	7.58E-01
20	23536	Triglycerides in Large LDL	mmol	Lipoprotein subclasses	-7.55E-01
21	23501	Triglycerides in Large VLDL	mmol	Lipoprotein subclasses	7.42E-01
22	23512	Cholesterol in Small VLDL	mmol	Lipoprotein subclasses	7.42E-01

Rank[a]	Field ID	Metabolites	Units	Group	Coefficients
23	23497	Phospholipids in Large VLDL	mmol	Lipoprotein subclasses	-7.08E-01
24	23421	Free Cholesterol in LDL	mmol	Free cholesterol	-6.77E-01
25	23515	Triglycerides in Small VLDL	mmol	Lipoprotein subclasses	6.71E-01
26	23447	Monounsaturated Fatty Acids	mmol	Fatty acids	-6.59E-01
27	23542	Free Cholesterol in Medium LDL	mmol	Lipoprotein subclasses	6.11E-01
28	23529	Triglycerides in IDL	mmol	Lipoprotein subclasses	5.74E-01
29	23558	Concentration of Large HDL Particles	mmol	Lipoprotein subclasses	5.65E-01
30	23499	Cholesteryl Esters in Large VLDL	mmol	Lipoprotein subclasses	5.55E-01
31	23487	Triglycerides in Chylomicrons and Extremely Large VLDL	mmol	Lipoprotein subclasses	-5.54E-01
32	23428	Concentration of VLDL Particles	mmol	Lipoprotein particle concentrations	-5.36E-01
33	23492	Cholesteryl Esters in Very Large VLDL	mmol	Lipoprotein subclasses	-5.34E-01
34	23549	Free Cholesterol in Small LDL	mmol	Lipoprotein subclasses	5.30E-01
35	23436	Total Cholines	mmol	Other lipids	5.14E-01
36	23484	Cholesterol in Chylomicrons and Extremely Large VLDL	mmol	Lipoprotein subclasses	5.02E-01
37	23527	Cholesteryl Esters in IDL	mmol	Lipoprotein subclasses	5.00E-01
38	23429	Concentration of LDL Particles	mmol	Lipoprotein particle concentrations	4.75E-01
39	23521	Free Cholesterol in Very Small VLDL	mmol	Lipoprotein subclasses	4.56E-01
40	23444	Omega-3 Fatty Acids	mmol	Fatty acids	4.54E-01
41	23513	Cholesteryl Esters in Small VLDL	mmol	Lipoprotein subclasses	4.41E-01
42	23504	Phospholipids in Medium VLDL	mmol	Lipoprotein subclasses	-4.31E-01

Rank[a]	Field ID	Metabolites	Units	Group	Coefficients
43	23425	Total Lipids in LDL	mmol	Total lipids	-4.17E-01
44	23488	Concentration of Very Large VLDL Particles	mmol	Lipoprotein subclasses	-4.15E-01
45	23412	Phospholipids in VLDL	mmol	Phospholipids	-4.11E-01
46	23569	Cholesteryl Esters in Medium HDL	mmol	Lipoprotein subclasses	-4.03E-01
47	23522	Triglycerides in Very Small VLDL	mmol	Lipoprotein subclasses	3.87E-01
48	23494	Triglycerides in Very Large VLDL	mmol	Lipoprotein subclasses	-3.77E-01
49	23524	Total Lipids in IDL	mmol	Lipoprotein subclasses	3.70E-01
50	23400	Total Cholesterol	mmol	Cholesterol	3.47E-01
51	23546	Phospholipids in Small LDL	mmol	Lipoprotein subclasses	-3.42E-01
52	23519	Cholesterol in Very Small VLDL	mmol	Lipoprotein subclasses	3.41E-01
53	23467	Valine	mmol	Amino acids	3.22E-01
54	23410	Triglycerides in HDL	mmol	Triglycerides	-3.20E-01
55	23433	Average Diameter for HDL Particles	nm	Lipoprotein particle sizes	3.00E-01
56	23544	Concentration of Small LDL Particles	mmol	Lipoprotein subclasses	2.87E-01
57	23414	Phospholipids in HDL	mmol	Phospholipids	2.82E-01
58	23413	Phospholipids in LDL	mmol	Phospholipids	-2.73E-01
59	23514	Free Cholesterol in Small VLDL	mmol	Lipoprotein subclasses	-2.72E-01
60	23566	Total Lipids in Medium HDL	mmol	Lipoprotein subclasses	-2.67E-01
61	23543	Triglycerides in Medium LDL	mmol	Lipoprotein subclasses	2.64E-01
62	23485	Cholesteryl Esters in Chylomicrons and Extremely Large VLDL	mmol	Lipoprotein subclasses	-2.61E-01
63	23505	Cholesterol in Medium VLDL	mmol	Lipoprotein subclasses	-2.50E-01
64	23434	Phosphoglycerides	mmol	Other lipids	-2.46E-01
65	23568	Cholesterol in Medium HDL	mmol	Lipoprotein subclasses	-2.34E-01
66	23520	Cholesteryl Esters in Very Small VLDL	mmol	Lipoprotein subclasses	-2.32E-01

Rank[a]	Field ID	Metabolites	Units	Group	Coefficients
67	23466	Leucine	mmol	Amino acids	-2.22E-01
68	23508	Triglycerides in Medium VLDL	mmol	Lipoprotein subclasses	2.19E-01
69	23480	Glycoprotein Acetyls	mmol	Inflammation	-2.15E-01
70	23402	Remnant Cholesterol (Non-HDL, Non-LDL - Cholesterol)	mmol	Cholesterol	2.13E-01
71	23534	Cholesteryl Esters in Large LDL	mmol	Lipoprotein subclasses	2.11E-01
72	23483	Phospholipids in Chylomicrons and Extremely Large VLDL	mmol	Lipoprotein subclasses	-2.09E-01
73	23530	Concentration of Large LDL Particles	mmol	Lipoprotein subclasses	2.08E-01
74	23567	Phospholipids in Medium HDL	mmol	Lipoprotein subclasses	2.08E-01
75	23438	Sphingomyelins	mmol	Other lipids	-2.06E-01
76	23493	Free Cholesterol in Very Large VLDL	mmol	Lipoprotein subclasses	2.03E-01
77	23431	Average Diameter for VLDL Particles	nm	Lipoprotein particle sizes	2.02E-01
78	23552	Total Lipids in Very Large HDL	mmol	Lipoprotein subclasses	-2.00E-01
79	23541	Cholesteryl Esters in Medium LDL	mmol	Lipoprotein subclasses	-1.98E-01
80	23526	Cholesterol in IDL	mmol	Lipoprotein subclasses	-1.96E-01
81	23407	Total Triglycerides	mmol	Triglycerides	1.95E-01
82	23506	Cholesteryl Esters in Medium VLDL	mmol	Lipoprotein subclasses	-1.83E-01
83	23563	Free Cholesterol in Large HDL	mmol	Lipoprotein subclasses	-1.82E-01
84	23486	Free Cholesterol in Chylomicrons and Extremely Large VLDL	mmol	Lipoprotein subclasses	1.81E-01
85	23473	Citrate	mmol	Glycolysis related metabolites	1.80E-01
86	23509	Concentration of Small VLDL Particles	mmol	Lipoprotein subclasses	1.74E-01
87	23446	Polyunsaturated Fatty Acids	mmol	Fatty acids	-1.72E-01

Rank[a]	Field ID	Metabolites	Units	Group	Coefficients
88	23474	3-Hydroxybutyrate	mmol	Ketone bodies	1.70E-01
89	23510	Total Lipids in Small VLDL	mmol	Lipoprotein subclasses	-1.67E-01
90	23476	Acetoacetate	mmol	Ketone bodies	-1.57E-01
91	23424	Total Lipids in VLDL	mmol	Total lipids	-1.52E-01
92	23409	Triglycerides in LDL	mmol	Triglycerides	1.51E-01
93	23532	Phospholipids in Large LDL	mmol	Lipoprotein subclasses	-1.51E-01
94	23423	Total Lipids in Lipoprotein Particles	mmol	Total lipids	1.48E-01
95	23462	Glycine	mmol	Amino acids	1.46E-01
96	23500	Free Cholesterol in Large VLDL	mmol	Lipoprotein subclasses	1.43E-01
97	23469	Tyrosine	mmol	Amino acids	1.41E-01
98	23503	Total Lipids in Medium VLDL	mmol	Lipoprotein subclasses	-1.37E-01
99	23495	Concentration of Large VLDL Particles	mmol	Lipoprotein subclasses	-1.36E-01
100	23538	Total Lipids in Medium LDL	mmol	Lipoprotein subclasses	-1.35E-01
101	23401	Total Cholesterol Minus HDL-C	mmol	Cholesterol	1.33E-01
102	23571	Triglycerides in Medium HDL	mmol	Lipoprotein subclasses	-1.31E-01
103	23565	Concentration of Medium HDL Particles	mmol	Lipoprotein subclasses	-1.28E-01
104	23416	Cholesteryl Esters in VLDL	mmol	Cholesteryl esters	-1.26E-01
105	23478	Creatinine	mmol	Fluid balance	-1.23E-01
106	23540	Cholesterol in Medium LDL	mmol	Lipoprotein subclasses	-1.22E-01
107	23564	Triglycerides in Large HDL	mmol	Lipoprotein subclasses	-1.13E-01
108	23481	Concentration of Chylomicrons and Extremely Large VLDL Particles	mmol	Lipoprotein subclasses	-1.01E-01
109	23516	Concentration of Very Small VLDL Particles	mmol	Lipoprotein subclasses	9.78E-02
110	23448	Saturated Fatty Acids	mmol	Fatty acids	9.30E-02
111	23557	Triglycerides in Very Large HDL	mmol	Lipoprotein subclasses	-8.95E-02

Rank[a]	Field ID	Metabolites	Units	Group	Coefficients
112	23461	Glutamine	mmol	Amino acids	-8.69E-02
113	23460	Alanine	mmol	Amino acids	-8.14E-02
114	23406	HDL Cholesterol	mmol	Cholesterol	7.19E-02
115	23489	Total Lipids in Very Large VLDL	mmol	Lipoprotein subclasses	6.74E-02
116	23442	Total Fatty Acids	mmol	Fatty acids	6.71E-02
117	23465	Isoleucine	mmol	Amino acids	-6.39E-02
118	23507	Free Cholesterol in Medium VLDL	mmol	Lipoprotein subclasses	5.78E-02
119	23418	Cholesteryl Esters in HDL	mmol	Cholesteryl esters	5.60E-02
120	23417	Cholesteryl Esters in LDL	mmol	Cholesteryl esters	-5.44E-02
121	23490	Phospholipids in Very Large VLDL	mmol	Lipoprotein subclasses	5.35E-02
122	23482	Total Lipids in Chylomicrons and Extremely Large VLDL	mmol	Lipoprotein subclasses	-4.71E-02
123	23537	Concentration of Medium LDL Particles	mmol	Lipoprotein subclasses	4.57E-02
124	23464	Total Concentration of Branched-Chain Amino Acids (Leucine + Isoleucine + Valine)	mmol	Amino acids	4.31E-02
125	23432	Average Diameter for LDL Particles	nm	Lipoprotein particle sizes	-3.39E-02
126	23420	Free Cholesterol in VLDL	mmol	Free cholesterol	3.35E-02
127	23468	Phenylalanine	mmol	Amino acids	-3.12E-02
128	23430	Concentration of HDL Particles	mmol	Lipoprotein particle concentrations	3.04E-02
129	23551	Concentration of Very Large HDL Particles	mmol	Lipoprotein subclasses	3.03E-02
130	23523	Concentration of IDL Particles	mmol	Lipoprotein subclasses	-2.90E-02
131	23560	Phospholipids in Large HDL	mmol	Lipoprotein subclasses	-2.80E-02
132	23559	Total Lipids in Large HDL	mmol	Lipoprotein subclasses	2.78E-02
133	23477	Acetone	mmol	Ketone bodies	-2.72E-02

Rank[a]	Field ID	Metabolites	Units	Group	Coefficients
134	23471	Lactate	mmol	Glycolysis related metabolites	-2.37E-02
135	23426	Total Lipids in HDL	mmol	Total lipids	-2.24E-02
136	23470	Glucose	mmol	Glycolysis related metabolites	2.21E-02
137	23450	Docosahexaenoic Acid	mmol	Fatty acids	-2.17E-02
138	23479	Albumin	g/l	Fluid balance	-1.89E-02
139	23411	Total Phospholipids in Lipoprotein Particles	mmol	Phospholipids	-1.68E-02
140	23576	Cholesteryl Esters in Small HDL	mmol	Lipoprotein subclasses	1.63E-02
141	23463	Histidine	mmol	Amino acids	-1.56E-02
142	23578	Triglycerides in Small HDL	mmol	Lipoprotein subclasses	1.46E-02
143	23517	Total Lipids in Very Small VLDL	mmol	Lipoprotein subclasses	-1.31E-02
144	23555	Cholesteryl Esters in Very Large HDL	mmol	Lipoprotein subclasses	1.26E-02
145	23472	Pyruvate	mmol	Glycolysis related metabolites	1.25E-02
146	23554	Cholesterol in Very Large HDL	mmol	Lipoprotein subclasses	1.22E-02
147	23475	Acetate	mmol	Ketone bodies	1.15E-02
148	23556	Free Cholesterol in Very Large HDL	mmol	Lipoprotein subclasses	-8.96E-03
149	23545	Total Lipids in Small LDL	mmol	Lipoprotein subclasses	-8.88E-03
150	23525	Phospholipids in IDL	mmol	Lipoprotein subclasses	8.81E-03
151	23440	Apolipoprotein A1	g/l	Apolipoproteins	8.19E-03
152	23439	Apolipoprotein B	g/l	Apolipoproteins	-5.70E-03
153	23547	Cholesterol in Small LDL	mmol	Lipoprotein subclasses	-2.94E-03
154	23531	Total Lipids in Large LDL	mmol	Lipoprotein subclasses	-2.67E-03
155	23539	Phospholipids in Medium LDL	mmol	Lipoprotein subclasses	2.30E-03
156	23415	Total Esterified Cholesterol	mmol	Cholesteryl esters	-2.01E-03
157	23405	LDL Cholesterol	mmol	Cholesterol	1.52E-03

Rank[a]	Field ID	Metabolites	Units	Group	Coefficients
158	23561	Cholesterol in Large HDL	mmol	Lipoprotein subclasses	-1.49E-03
159	23548	Cholesteryl Esters in Small LDL	mmol	Lipoprotein subclasses	7.67E-04
160	23496	Total Lipids in Large VLDL	mmol	Lipoprotein subclasses	5.50E-04
161	23572	Concentration of Small HDL Particles	mmol	Lipoprotein subclasses	-4.05E-04
162	23533	Cholesterol in Large LDL	mmol	Lipoprotein subclasses	3.45E-04
163	23491	Cholesterol in Very Large VLDL	mmol	Lipoprotein subclasses	-3.05E-05
164	23408	Triglycerides in VLDL	mmol	Triglycerides	.
164	23570	Free Cholesterol in Medium HDL	mmol	Lipoprotein subclasses	.
164	23403	VLDL Cholesterol	mmol	Cholesterol	.
164	23404	Clinical LDL Cholesterol	mmol	Cholesterol	.

[a] Metabolites are ranked by the absolute value of the coefficients in descending order. The top 25 metabolites range from Total Free Cholesterol to Triglycerides in Small VLDL. Abbreviations: LDL, low-density lipoprotein cholesterol; HDL, high-density lipoprotein cholesterol; VLDL, very low-density lipoprotein.

Table S3. Risk of Premature Mortality according to Different Categories of Metabolomic Signature

Metabolic status	No. of events	Person years	Model 1[a]		Model 2 [a]		Model 3 [a]	
			HR (95%CI)	P value	HR (95%CI)	P value	HR (95%CI)	P value
Based on quartiles of metabolomic signature[b]								
Low	565	110,801	1.84 (1.61,2.12)	<0.001	1.84 (1.60,2.11)	<0.001	1.65 (1.42,1.91)	<0.001
Intermediate	734	225,400	1.18 (1.03,1.35)	0.015	1.20 (1.05,1.37)	0.007	1.18 (1.03,1.35)	0.020
High	313	113,394	(Reference)		(Reference)		(Reference)	
P value for trend			<0.001		<0.001		<0.001	
Based on quintile of metabolomic signature[c]								
Low	502	88,188	2.06 (1.77,2.40)	<0.001	2.05 (1.76,2.39)	<0.001	1.82 (1.55,2.14)	<0.001
Intermediate	860	270,667	1.15 (1.00,1.33)	0.048	1.17 (1.02,1.35)	0.030	1.14 (0.98,1.31)	0.089
High	250	90,740	(Reference)		(Reference)		(Reference)	
P value for trend			<0.001		<0.001		<0.001	

[a] Model 1 was not adjusted; model 2 was adjusted for age, race; model 3 was further adjusted for education, employment, BMI, healthy alcohol intake, healthy diet, healthy physical activity, and menopause hormone therapy.

[b] Defined by quartiles of metabolomic signature: low (the bottom quartile), intermediate (quartiles 2-3) and high (the top quartile).

[c] Defined by quintiles of metabolomic signature: low (the bottom quintile), intermediate (quintiles 2-4) and high (the top quintile).

Abbreviations: HR, hazards ratio; CI, confidence interval.

Table S4. Cox Regression Analyses of Age at Menopause and Cause-specific Premature Mortality

Cause-specific premature mortality	Model 1[a]		Model 2 [a]		Model 3 [a]	
	HR (95%CI)	P value	HR (95%CI)	P value	HR (95%CI)	P value
Type 2 diabetes	0.98 (0.94,1.02)	0.260	0.97 (0.93,1.01)	0.120	0.98 (0.94,1.03)	0.409
Hypertension	0.97 (0.94,0.99)	0.020	0.96 (0.94,0.99)	0.006	0.97 (0.94,1.00)	0.053
Cardiovascular disease	0.97 (0.95,0.99)	0.018	0.96 (0.94,0.99)	0.002	0.97 (0.95,1.00)	0.032
Chronic kidney disease	0.93 (0.90,0.96)	< 0.001	0.92 (0.89,0.95)	< 0.001	0.93 (0.90,0.97)	< 0.001
Chronic liver disease	0.91 (0.86,0.97)	0.004	0.91 (0.86,0.97)	0.006	0.91 (0.86,0.97)	0.007
Cancer	0.98 (0.94,1.02)	0.403	0.98 (0.94,1.02)	0.284	0.98 (0.94,1.03)	0.465
All Other Causes	0.98 (0.97,0.99)	< 0.001	0.98 (0.97,0.99)	< 0.001	0.98 (0.97,0.99)	< 0.001

[a] Model 1 was not adjusted; model 2 was adjusted for age, race; model 3 was further adjusted for education, employment, BMI, healthy alcohol intake, healthy diet, healthy physical activity, and menopause hormone therapy.
Abbreviations: HR, hazards ratio; CI, confidence interval.

Table S5. Associations of Age at Menopause and Metabolomic Signature with Premature Mortality by Varying Age Cut-off Points

	Model 1[a]	Model 2[a]	Model 3[a]
		**Death Before Age 65**	
Age at menopause (continuous)	0.94 (0.93,0.96)	0.97 (0.96,0.99)	0.98 (0.97,1.00)
Age at menopause group			
Menopause age ≥50	Reference	Reference	Reference
Menopause age 40-49	1.75 (1.43,2.14)	1.25 (1.01,1.55)	1.23 (0.98,1.55)
Menopause age <40	2.60 (1.80,3.75)	1.50 (1.02,2.21)	1.41 (0.90,2.21)
Metabolomic signature (continuous)	0.64 (0.55,0.73)	0.63 (0.54,0.73)	0.65 (0.55,0.77)
Metabolomic signature group			
High metabolomic signature	Reference	Reference	Reference
Intermediate metabolomic signature	1.49 (1.13, 1.97)	1.31 (0.99, 1.72)	1.28 (0.95, 1.71)
Low metabolomic signature	2.19 (1.64, 2.93)	2.05 (1.53, 2.74)	1.93 (1.41, 2.63)
		**Death Before Age 70**	
Age at menopause (continuous)	0.97 (0.96,0.98)	0.97 (0.96,0.99)	0.98 (0.97,1.00)
Age at menopause group			
Menopause age ≥50	Reference	Reference	Reference
Menopause age 40-49	1.27 (1.11,1.46)	1.17 (1.02,1.35)	1.11 (0.96,1.29)
Menopause age <40	1.90 (1.46,2.47)	1.68 (1.29,2.20)	1.43 (1.06,1.92)
Metabolomic signature (continuous)	0.66 (0.60,0.73)	0.65 (0.59,0.72)	0.69 (0.62,0.77)
Metabolomic signature group			
High metabolomic signature	Reference	Reference	Reference
Intermediate metabolomic signature	1.33 (1.11, 1.59)	1.28 (1.07, 1.53)	1.29 (1.07, 1.56)
Low metabolomic signature	1.88 (1.56, 2.28)	1.88 (1.55, 2.27)	1.72 (1.40, 2.11)

		**Death Before Age 75**	
Age at menopause (continuous)	0.98 (0.97,0.98)	0.97 (0.96,0.98)	0.98 (0.97,0.99)
Age at menopause group			
Menopause age ≥50	Reference	Reference	Reference
Menopause age 40-49	1.16 (1.05,1.29)	1.22 (1.10,1.36)	1.17 (1.04,1.30)
Menopause age <40	1.64 (1.34,2.02)	1.76 (1.43,2.17)	1.60 (1.28,2.00)
Metabolomic signature (continuous)	0.65 (0.60,0.70)	0.65 (0.61,0.70)	0.70 (0.65,0.76)
Metabolomic signature group			
High metabolomic signature	Reference	Reference	Reference
Intermediate metabolomic signature	1.18 (1.03, 1.35)	1.20 (1.05, 1.37)	1.18 (1.03, 1.35)
Low metabolomic signature	1.84 (1.61, 2.12)	1.84 (1.60, 2.11)	1.65 (1.42, 1.91)

[a] Model 1 was not adjusted; model 2 was adjusted for age, race; model 3 was further adjusted for education, employment, BMI, healthy alcohol intake, healthy diet, healthy physical activity, and menopause hormone therapy.

Table S6. Metabolites Exclusively Correlated with Age at Menopause, Premature Mortality, and Their Intersection

Field ID	Metabolites	Group	Age at Menopause Metabolites [a]	Premature Mortality Metabolites [b]	Metabolites Intersect Both
23444	Omega-3 Fatty Acids	Fatty acids	No	No	Yes
23450	Docosahexaenoic Acid	Fatty acids	No	No	Yes
23551	Concentration of Very Large HDL Particles	Lipoprotein subclasses	No	No	Yes
23555	Cholesteryl Esters in Very Large HDL	Lipoprotein subclasses	No	No	Yes
23552	Total Lipids in Very Large HDL	Lipoprotein subclasses	No	No	Yes
23554	Cholesterol in Very Large HDL	Lipoprotein subclasses	No	No	Yes
23553	Phospholipids in Very Large HDL	Lipoprotein subclasses	No	No	Yes
23558	Concentration of Large HDL Particles	Lipoprotein subclasses	No	No	Yes
23563	Free Cholesterol in Large HDL	Lipoprotein subclasses	No	No	Yes
23433	Average Diameter for HDL Particles	Lipoprotein particle sizes	No	No	Yes
23446	Polyunsaturated Fatty Acids	Fatty acids	No	No	Yes
23556	Free Cholesterol in Very Large HDL	Lipoprotein subclasses	No	No	Yes
23561	Cholesterol in Large HDL	Lipoprotein subclasses	No	No	Yes
23562	Cholesteryl Esters in Large HDL	Lipoprotein subclasses	No	No	Yes
23559	Total Lipids in Large HDL	Lipoprotein subclasses	No	No	Yes
23560	Phospholipids in Large HDL	Lipoprotein subclasses	No	No	Yes
23520	Cholesteryl Esters in Very Small VLDL	Lipoprotein subclasses	No	No	Yes
23422	Free Cholesterol in HDL	Free cholesterol	No	No	Yes
23528	Free Cholesterol in IDL	Lipoprotein subclasses	No	No	Yes
23519	Cholesterol in Very Small VLDL	Lipoprotein subclasses	No	No	Yes

23525	Phospholipids in IDL	Lipoprotein subclasses	No	No	Yes
23419	Total Free Cholesterol	Free cholesterol	No	No	Yes
23524	Total Lipids in IDL	Lipoprotein subclasses	No	No	Yes
23526	Cholesterol in IDL	Lipoprotein subclasses	No	No	Yes
23400	Total Cholesterol	Cholesterol	No	No	Yes
23564	Triglycerides in Large HDL	Lipoprotein subclasses	Yes	No	No
23415	Total Esterified Cholesterol	Cholesteryl esters	No	No	Yes
23527	Cholesteryl Esters in IDL	Lipoprotein subclasses	No	No	Yes
23437	Phosphatidylcholines	Other lipids	No	No	Yes
23438	Sphingomyelins	Other lipids	No	No	Yes
23521	Free Cholesterol in Very Small VLDL	Lipoprotein subclasses	Yes	No	No
23411	Total Phospholipids in Lipoprotein Particles	Phospholipids	No	No	Yes
23406	HDL Cholesterol	Cholesterol	No	No	Yes
23480	Glycoprotein Acetyls	Inflammation	No	No	Yes
23436	Total Cholines	Other lipids	No	No	Yes
23557	Triglycerides in Very Large HDL	Lipoprotein subclasses	No	No	Yes
23506	Cholesteryl Esters in Medium VLDL	Lipoprotein subclasses	No	No	Yes
23426	Total Lipids in HDL	Total lipids	No	No	Yes
23517	Total Lipids in Very Small VLDL	Lipoprotein subclasses	Yes	No	No
23418	Cholesteryl Esters in HDL	Cholesteryl esters	No	No	Yes
23434	Phosphoglycerides	Other lipids	No	No	Yes
23516	Concentration of Very Small VLDL Particles	Lipoprotein subclasses	Yes	No	No
23505	Cholesterol in Medium VLDL	Lipoprotein subclasses	No	No	Yes

23535	Free Cholesterol in Large LDL	Lipoprotein subclasses	No	No	Yes
23414	Phospholipids in HDL	Phospholipids	No	No	Yes
23462	Glycine	Amino acids	No	No	Yes
23402	Remnant Cholesterol (Non-HDL, Non-LDL -Cholesterol)	Cholesterol	No	No	Yes
23423	Total Lipids in Lipoprotein Particles	Total lipids	No	No	Yes
23518	Phospholipids in Very Small VLDL	Lipoprotein subclasses	Yes	No	No
23533	Cholesterol in Large LDL	Lipoprotein subclasses	No	No	Yes
23570	Free Cholesterol in Medium HDL	Lipoprotein subclasses	No	No	Yes
23523	Concentration of IDL Particles	Lipoprotein subclasses	No	No	Yes
23421	Free Cholesterol in LDL	Free cholesterol	No	No	Yes
23401	Total Cholesterol Minus HDL-C	Cholesterol	No	No	Yes
23534	Cholesteryl Esters in Large LDL	Lipoprotein subclasses	No	No	Yes
23531	Total Lipids in Large LDL	Lipoprotein subclasses	No	No	Yes
23440	Apolipoprotein A1	Apolipoproteins	No	No	Yes
23479	Albumin	Fluid balance	No	No	Yes
23439	Apolipoprotein B	Apolipoproteins	No	No	Yes
23429	Concentration of LDL Particles	Lipoprotein particle concentrations	No	No	Yes
23404	Clinical LDL Cholesterol	Cholesterol	No	No	Yes
23532	Phospholipids in Large LDL	Lipoprotein subclasses	No	No	Yes
23507	Free Cholesterol in Medium VLDL	Lipoprotein subclasses	No	No	Yes
23537	Concentration of Medium LDL Particles	Lipoprotein subclasses	No	No	Yes
23530	Concentration of Large LDL Particles	Lipoprotein subclasses	No	No	Yes

23449	Linoleic Acid	Fatty acids	No	No	Yes
23405	LDL Cholesterol	Cholesterol	No	No	Yes
23544	Concentration of Small LDL Particles	Lipoprotein subclasses	No	No	Yes
23549	Free Cholesterol in Small LDL	Lipoprotein subclasses	No	No	Yes
23417	Cholesteryl Esters in LDL	Cholesteryl esters	No	No	Yes
23514	Free Cholesterol in Small VLDL	Lipoprotein subclasses	No	No	Yes
23445	Omega-6 Fatty Acids	Fatty acids	No	No	Yes
23425	Total Lipids in LDL	Total lipids	No	No	Yes
23427	Total Concentration of Lipoprotein Particles	Lipoprotein particle concentrations	No	No	Yes
23416	Cholesteryl Esters in VLDL	Cholesteryl esters	No	No	Yes
23542	Free Cholesterol in Medium LDL	Lipoprotein subclasses	No	No	Yes
23565	Concentration of Medium HDL Particles	Lipoprotein subclasses	No	No	Yes
23473	Citrate	Glycolysis related metabolites	Yes	No	No
23413	Phospholipids in LDL	Phospholipids	No	No	Yes
23469	Tyrosine	Amino acids	Yes	No	No
23546	Phospholipids in Small LDL	Lipoprotein subclasses	No	No	Yes
23504	Phospholipids in Medium VLDL	Lipoprotein subclasses	No	No	Yes
23568	Cholesterol in Medium HDL	Lipoprotein subclasses	No	No	Yes
23430	Concentration of HDL Particles	Lipoprotein particle concentrations	No	No	Yes
23475	Acetate	Ketone bodies	No	No	Yes
23511	Phospholipids in Small VLDL	Lipoprotein subclasses	Yes	No	No
23512	Cholesterol in Small VLDL	Lipoprotein subclasses	Yes	No	No

23547	Cholesterol in Small LDL	Lipoprotein subclasses	No	No	Yes
23431	Average Diameter for VLDL Particles	Lipoprotein particle sizes	No	No	Yes
23545	Total Lipids in Small LDL	Lipoprotein subclasses	No	No	Yes
23566	Total Lipids in Medium HDL	Lipoprotein subclasses	No	No	Yes
23478	Creatinine	Fluid balance	No	No	Yes
23540	Cholesterol in Medium LDL	Lipoprotein subclasses	No	No	Yes
23403	VLDL Cholesterol	Cholesterol	Yes	No	No
23502	Concentration of Medium VLDL Particles	Lipoprotein subclasses	No	No	Yes
23569	Cholesteryl Esters in Medium HDL	Lipoprotein subclasses	No	No	Yes
23467	Valine	Amino acids	No	No	Yes
23548	Cholesteryl Esters in Small LDL	Lipoprotein subclasses	No	No	Yes
23541	Cholesteryl Esters in Medium LDL	Lipoprotein subclasses	No	No	Yes
23428	Concentration of VLDL Particles	Lipoprotein particle concentrations	Yes	No	No
23538	Total Lipids in Medium LDL	Lipoprotein subclasses	No	No	Yes
23567	Phospholipids in Medium HDL	Lipoprotein subclasses	No	No	Yes
23442	Total Fatty Acids	Fatty acids	Yes	No	No
23513	Cholesteryl Esters in Small VLDL	Lipoprotein subclasses	Yes	No	No
23503	Total Lipids in Medium VLDL	Lipoprotein subclasses	No	No	Yes
23483	Phospholipids in Chylomicrons and Extremely Large VLDL	Lipoprotein subclasses	No	No	Yes
23576	Cholesteryl Esters in Small HDL	Lipoprotein subclasses	No	Yes	No
23487	Triglycerides in Chylomicrons and Extremely Large VLDL	Lipoprotein subclasses	No	Yes	No

23482	Total Lipids in Chylomicrons and Extremely Large VLDL	Lipoprotein subclasses	No	Yes	No
23470	Glucose	Glycolysis related metabolites	No	No	No
23529	Triglycerides in IDL	Lipoprotein subclasses	No	Yes	No
23578	Triglycerides in Small HDL	Lipoprotein subclasses	No	Yes	No
23472	Pyruvate	Glycolysis related metabolites	No	Yes	No
23510	Total Lipids in Small VLDL	Lipoprotein subclasses	No	No	No
23481	Concentration of Chylomicrons and Extremely Large VLDL Particles	Lipoprotein subclasses	No	Yes	No
23484	Cholesterol in Chylomicrons and Extremely Large VLDL	Lipoprotein subclasses	No	Yes	No
23486	Free Cholesterol in Chylomicrons and Extremely Large VLDL	Lipoprotein subclasses	No	Yes	No
23539	Phospholipids in Medium LDL	Lipoprotein subclasses	No	Yes	No
23485	Cholesteryl Esters in Chylomicrons and Extremely Large VLDL	Lipoprotein subclasses	No	Yes	No
23420	Free Cholesterol in VLDL	Free cholesterol	No	No	No
23509	Concentration of Small VLDL Particles	Lipoprotein subclasses	No	No	No
23448	Saturated Fatty Acids	Fatty acids	No	No	No
23577	Free Cholesterol in Small HDL	Lipoprotein subclasses	No	Yes	No
23447	Monounsaturated Fatty Acids	Fatty acids	No	Yes	No
23573	Total Lipids in Small HDL	Lipoprotein subclasses	No	No	No
23494	Triglycerides in Very Large VLDL	Lipoprotein subclasses	No	Yes	No
23476	Acetoacetate	Ketone bodies	No	Yes	No
23572	Concentration of Small HDL Particles	Lipoprotein subclasses	No	Yes	No

23464	Total Concentration of Branched-Chain Amino Acids (Leucine + Isoleucine + Valine)	Amino acids	No	Yes	No
23522	Triglycerides in Very Small VLDL	Lipoprotein subclasses	No	Yes	No
23536	Triglycerides in Large LDL	Lipoprotein subclasses	No	Yes	No
23499	Cholesteryl Esters in Large VLDL	Lipoprotein subclasses	No	No	No
23574	Phospholipids in Small HDL	Lipoprotein subclasses	No	No	No
23575	Cholesterol in Small HDL	Lipoprotein subclasses	No	Yes	No
23488	Concentration of Very Large VLDL Particles	Lipoprotein subclasses	No	Yes	No
23412	Phospholipids in VLDL	Phospholipids	No	No	No
23409	Triglycerides in LDL	Triglycerides	No	Yes	No
23410	Triglycerides in HDL	Triglycerides	No	Yes	No
23489	Total Lipids in Very Large VLDL	Lipoprotein subclasses	No	Yes	No
23490	Phospholipids in Very Large VLDL	Lipoprotein subclasses	No	Yes	No
23497	Phospholipids in Large VLDL	Lipoprotein subclasses	No	Yes	No
23543	Triglycerides in Medium LDL	Lipoprotein subclasses	No	Yes	No
23424	Total Lipids in VLDL	Total lipids	No	No	No
23477	Acetone	Ketone bodies	No	Yes	No
23515	Triglycerides in Small VLDL	Lipoprotein subclasses	No	Yes	No
23550	Triglycerides in Small LDL	Lipoprotein subclasses	No	Yes	No
23468	Phenylalanine	Amino acids	No	Yes	No
23465	Isoleucine	Amino acids	No	No	No
23408	Triglycerides in VLDL	Triglycerides	No	Yes	No
23493	Free Cholesterol in Very Large VLDL	Lipoprotein subclasses	No	Yes	No

23500	Free Cholesterol in Large VLDL	Lipoprotein subclasses	No	No	No
23461	Glutamine	Amino acids	No	Yes	No
23466	Leucine	Amino acids	No	Yes	No
23495	Concentration of Large VLDL Particles	Lipoprotein subclasses	No	No	No
23508	Triglycerides in Medium VLDL	Lipoprotein subclasses	No	No	No
23498	Cholesterol in Large VLDL	Lipoprotein subclasses	No	No	No
23571	Triglycerides in Medium HDL	Lipoprotein subclasses	No	Yes	No
23501	Triglycerides in Large VLDL	Lipoprotein subclasses	No	No	No
23474	3-Hydroxybutyrate	Ketone bodies	No	Yes	No
23496	Total Lipids in Large VLDL	Lipoprotein subclasses	No	No	No
23463	Histidine	Amino acids	No	Yes	No
23492	Cholesteryl Esters in Very Large VLDL	Lipoprotein subclasses	No	No	No
23460	Alanine	Amino acids	No	No	No
23407	Total Triglycerides	Triglycerides	No	Yes	No
23491	Cholesterol in Very Large VLDL	Lipoprotein subclasses	No	No	No
23471	Lactate	Glycolysis related metabolites	No	Yes	No
23432	Average Diameter for LDL Particles	Lipoprotein particle sizes	No	Yes	No

[a] Metabolites associated exclusively with age at menopause.
[b] Metabolites associated exclusively with premature mortality.
Abbreviations: LDL, low-density lipoprotein cholesterol; HDL, high-density lipoprotein cholesterol; VLDL, very low-density lipoprotein.

References

1. Elliott P, Peakman TC, UK Biobank. The UK Biobank sample handling and storage protocol for the collection, processing and archiving of human blood and urine. *Int J Epidemiol*. 2008;37(2):234-244.

2. Julkunen H, Cichońska A, Tiainen M, et al. Atlas of plasma NMR biomarkers for health and disease in 118,461 individuals from the UK Biobank. *Nat Commun*. 2023;14(1):604.

3. Lourida I, Hannon E, Littlejohns TJ, et al. Association of Lifestyle and Genetic Risk With Incidence of Dementia. *JAMA*. 2019;322(5):430.

4. Pazoki R, Dehghan A, Evangelou E, et al. Genetic Predisposition to High Blood Pressure and Lifestyle Factors: Associations With Midlife Blood Pressure Levels and Cardiovascular Events. *Circulation*. 2018;137(7):653-661.

5. Hagströmer M, Oja P, Sjöström M. The International Physical Activity Questionnaire (IPAQ): a study of concurrent and construct validity. *Public Health Nutr*. 2006;9(6):755-762.

6. Grambsch PM, Therneau TM. Proportional Hazards Tests and Diagnostics Based on Weighted Residuals. *Biometrika*. 1994;81(3):515-526.

7. Desquilbet L, Mariotti F. Dose-response analyses using restricted cubic spline functions in public health research. *Stat Med*. 2010;29(9):1037-1057.

8. Durrleman S, Simon R. Flexible regression models with cubic splines. *Stat Med*. 1989;8(5):551-561.

9. Harrell , FE. *Regression Modeling Strategies: With Applications to Linear Models, Logistic and Ordinal Regression, and Survival Analysis*. Springer International Publishing; 2015.

10. Harrell. F. R rms Package. Hbiostat.org. Updated September 26, 2023. Accessed August 1, 2024. https://hbiostat.org/r/rms/

11. Baron RM, Kenny DA. The moderator-mediator variable distinction in social psychological research: conceptual, strategic, and statistical considerations. *J Pers Soc Psychol*. 1986;51(6):1173-1182.

12. Mackinnon DP, Warsi G, Dwyer JH. A Simulation Study of Mediated Effect Measures. *Multivar Behav Res*. 1995;30(1):41.

13. Albert JM. Mediation analysis via potential outcomes models. *Stat Med*. 2008;27(8):1282-1304.

14. Have TRT, Joffe MM, Lynch KG, Brown GK, Maisto SA, Beck AT. Causal mediation analyses with rank preserving models. *Biometrics*. 2007;63(3):926-934.

15. Robins JM, Greenland S. Identifiability and exchangeability for direct and indirect effects. *Epidemiol Camb Mass*. 1992;3(2):143-155.
